# Age-related evaluation of skin barrier parameters in retrievers

**DOI:** 10.3389/fvets.2025.1717581

**Published:** 2026-01-12

**Authors:** Eun-Hae Lee, Dongkuk Yoo, Ju-Ri Lee, Ji-Seon Kim, Hyun-Jung Han, Jae-Eun Hyun

**Affiliations:** 1 Department of Veterinary Internal Medicine, College of Veterinary Medicine, Konkuk University, Seoul, Republic of Korea; 2 Department of Veterinary Emergency and Critical Care, College of Veterinary Medicine, Konkuk University, Seoul, Republic of Korea; 3 KU Center for Animal Blood Medical Science, Konkuk University, Seoul, Republic of Korea

**Keywords:** Retrievers, canine skin barrier, aging effects, sebum, skin hydration, skin pH, transepidermal water loss

## Abstract

**Introduction:**

A disruption in the skin barrier, which serves as the primary defense against external pathogens, can lead to skin disorders. In humans, various factors influence the skin barrier, including age and anatomical site. Research on the canine skin barrier is limited; this study evaluated its function across age groups and anatomical sites in healthy retrievers.

**Methods:**

Forty-five healthy retrievers were included and divided into three age groups: young (2–3 years, *n* = 15), middle-aged (4–6 years, *n* = 15), and old (7–8 years, *n* = 15). The skin barrier parameters, including pH, transepidermal water loss (TEWL), skin hydration, sebum, and surface temperature, were measured at the ear pinnae, axillae, and inguinal region. Statistical analyses were used to compare parameters by age and site and to assess correlations between them.

**Results:**

Skin hydration significantly decreased with age at all anatomical sites. The pH and sebum levels varied by site, with the inguinal region showing the highest pH value and the pinnae showing the highest sebum and hydration levels. Negative correlations were found between TEWL and hydration and between TEWL and sebum. Meanwhile, positive correlations were observed between TEWL and temperature and between sebum and hydration.

**Conclusion:**

Skin hydration decreased with age and varied by anatomical site. The pH and sebum levels showed site-specific differences. The significant correlations between parameters highlight physiological interactions in skin barrier function. These baseline data provide a useful reference for dermatological evaluation and a foundation for future studies on canine skin barrier physiology.

## 1 Introduction

The skin acts as the main defensive barrier against physical, chemical, and microbial threats, and impaired skin barrier function has been associated with the development of various dermatological disorders (1–3). In particular, the stratum corneum plays an important role in limiting transepidermal water loss (TEWL), preventing external substance penetration, and maintaining homeostasis through the surface lipid film (4, 5). Several skin barrier parameters have been used to assess the skin barrier structure and function, including TEWL, skin hydration, skin pH, sebum, and temperature. In human studies, these parameters vary depending on several factors, including age, sex, anatomical site, and ethnicity (6–9). In addition, environmental conditions, including temperature, humidity, and air pollution, have been shown to influence skin barrier function (10, 11). Furthermore, previous studies have documented alterations in skin barrier parameters in various dermatological disorders. These include atopic dermatitis, psoriasis, and allergic contact dermatitis (12–14).

In dogs, several studies have reported differences in skin parameters related to anatomical site, breed, age, and sex (3, 15–17). However, most studies have focused on single parameters or limited anatomical sites. Meanwhile, investigations encompassing multiple sites, parameters, and age groups remain limited. Moreover, existing studies have primarily examined alterations in disease states, and baseline data from healthy dogs remain insufficient (1, 12). Establishing normative reference values in healthy individuals is essential for accurately interpreting skin barrier dysfunction observed in dermatological diseases. Nevertheless, studies comparing these parameters within a single breed are limited, and comprehensive evaluations across multiple anatomical sites and age groups in healthy dogs remain scarce.

Therefore, the present study aimed to noninvasively evaluate TEWL, skin pH, hydration, sebum, and temperature across different age groups and anatomical sites in healthy retrievers and to analyze the correlations between these parameters.

## 2 Materials and methods

### 2.1 Study population and demographics

Forty-five healthy retrievers (nine golden retrievers and 36 labrador retrievers) were enrolled from the KU I’M DOgNOR Blood Donation Center (Konkuk University, Seoul, Republic of Korea) between November 2023 and November 2024. The dogs were categorized into three age groups: young (2–3 years, average age 2.6 ± 0.49 years, *n* = 15), middle-aged (4–6 years, 4.6 ± 0.7 years, *n* = 15), and old (7–8 years, 7.53 ± 0.5 years, *n* = 15) (Table 1). All dogs were confirmed to be clinically healthy on the basis of physical examination, hematology, and serum biochemistry. Moreover, they had no history of skin disease, and no clinically apparent lesions were observed at the measurement sites. Dogs with diseases affecting the skin barrier, including endocrine disorders or malignant tumors, and those that were treated with topical or systemic antimicrobials within 2 weeks or glucocorticoids within 4 weeks before the study were excluded. The use of shampoo, ear cleansers, or topical agents was not permitted within 24 h before measurement and all dogs were client-owned. The present study was approved by the Institutional Animal Care and Use Committee of Konkuk University (approval no. KU25030).

**Table 1:** Age distribution of study subjects.

Parameter	Young(*n*=15)	Middle(*n*=15)	Old(*n*=15)
Mean ± SD (year)	2.6 ± 0.49	4.6 ± 0.7	7.5 ± 0.5
Min (year)	2	4	7
Max (year)	3	6	8

### 2.2 Measurement of skin barrier parameters

The following anatomical sites were used to measure skin barrier parameters: concave side of the ear pinnae, axillae, and inguinal region on either the left or right side. The least-haired area of each site was selected for measurement to minimize the influence of hair. Moreover, clipping was not performed to avoid skin hydration alterations. All measurements were performed by the same investigator in a quiet indoor environment maintained at a stable temperature of 20–24 °C and relative humidity of 40–65%. Before the measurement, the dogs were allowed a 15-min acclimatization period in the examination room (18) and restrained in lateral recumbency or standing position. All probes were gently placed perpendicular to the skin surface in accordance with the manufacturers’ instructions. The skin surface pH was measured using the Skin-pH-Meter PH 905 (Courage-Khazaka, Germany) and expressed in pH units. Meanwhile, TEWL was measured with an evaporimeter (VapoMeter SWL-3, Delfin Technologies Ltd., Finland) in accordance with established guidelines (19). The results were expressed as the evaporation rate (g/m^2^/h). Skin hydration was assessed using the Corneometer CM 825 (Courage-Khazaka GmbH, Germany) and expressed in arbitrary units (a.u.). Sebum levels were measured using the Sebumeter SM 815 (Courage-Khazaka GmbH, Germany) and expressed in μg/cm^2^. Sebum measurements were repeated at adjacent sites within the same area to minimize the absorption effect of probe contact. The skin surface temperature was recorded with a noncontact infrared thermometer (FS-300, HuBDIC, Korea) at a distance of 2–3 cm from the skin surface in accordance with the manufacturer’s instructions (20). All parameters were measured five times at each site, and the mean values were used for analysis.

### 2.3 Statistical analysis

The Shapiro–Wilk test was used to assess normality. Given that the data were not normally distributed, the Kruskal–Wallis H test followed by Dunn’s *post hoc* test was used to compare skin barrier parameters (i.e., TEWL, skin hydration, sebum, pH, and temperature) among the three age groups and three anatomical sites. Pearson’s correlation coefficient was used to evaluate correlations among skin barrier parameters. Correlation coefficients (*r*) < −0.2 and >0.2 were considered weak negative and positive correlations, respectively, whereas *r*-values <−0.4 and >0.4 indicated significant negative and positive correlations, respectively. Data were presented as median and interquartile range (IQR). Statistical analyses were performed using SPSS version 26.0 (IBM Corporation, USA) and GraphPad Prism version 10 (GraphPad Software, USA). Statistical significance was considered at *p* < 0.05.

## 3 Results

### 3.1 Age-related differences in skin barrier parameters

**Figure 1:**
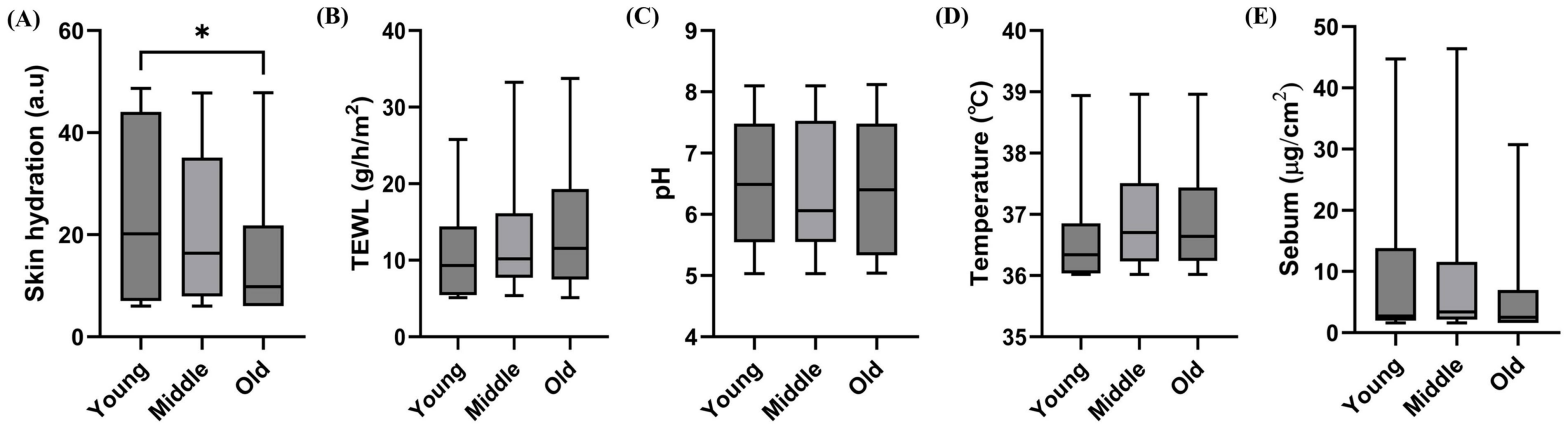
Variations in skin parameters across different age groups. **(A)** Skin hydration, **(B)** transepidermal water loss, **(C)** pH, **(D)** skin temperature, and **(E)** sebum. The box and whisker plots show the median, interquartile range, and minimum–maximum values. Statistical analysis was performed using the Kruskal–Wallis test followed by Dunn’s *post hoc* test (*p < 0.05).

The median (IQR) skin hydration levels (a.u.) were 20.20 (7.00–44.07), 16.40 (7.91–35.10), and 9.80 (6.00–21.80) in the young, middle-aged, and old groups, respectively, with significantly lower values in the old group than in the young and middle-aged groups (*p* = 0.034, Figure 1A). Meanwhile, the median (IQR) TEWL values (g/m^2^/h) were 9.32 (5.44–14.40), 10.18 (7.73–16.16), and 11.54 (7.47–19.31) in the young, middle-aged, and old groups, respectively. Although no significant differences were observed, an increasing trend with age was noted (*p* = 0.156, Figure 1B). The median (IQR) pH values were 6.49 (5.55–7.48), 6.06 (5.55–7.53), and 6.40 (5.33–7.48) in the young, middle-aged, and old groups, respectively, and no significant differences were observed (*p* = 0.925, Figure 1C). The median (IQR) temperature values were 36.34 (36.04–36.85), 36.70 (36.23–37.51), and 36.64 (36.24–37.44) in the young, middle-aged, and old groups, respectively, and no significant differences were observed (*p* = 0.062, Figure 1D). The median (IQR) sebum levels (μg/cm^2^) were 2.72 (1.96–13.80), 3.40 (2.14–11.58), and 2.52 (1.60–6.96) in the young, middle-aged, and old groups, respectively, and no significant differences were observed (*p* = 0.308, Figure 1E). Among the age groups, a significant difference was observed only in skin hydration, and TEWL tended to increase with age.

### 3.2 Site-related differences in skin barrier parameters

**Figure 2:**
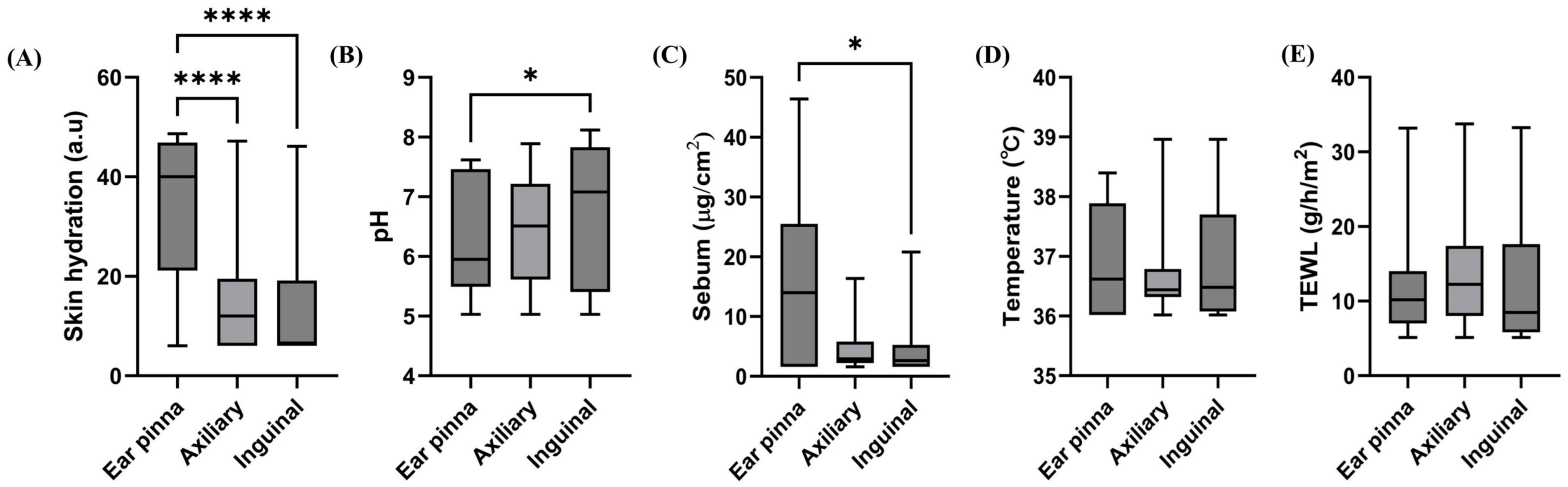
Variations in skin parameters across different anatomic sites. **(A)** Skin hydration, **(B)** pH, **(C)** sebum, **(D)** skin temperature, and **(E)** transepidermal water loss. The box and whisker plots show the median, interquartile range, and minimum–maximum values. Statistical analysis was performed using the Kruskal–Wallis test followed by Dunn’s *post hoc* test (*p < 0.05 and ****p < 0.0001).

The median (IQR) skin hydration levels (a.u.) were 40.00 (21.16–46.87), 12.00 (6.00–19.50), and 6.60 (6.00–19.20) at the ear pinnae, axillae, and inguinal region, respectively. Significant differences were observed among sites, and the highest values were found at the ear pinnae (*p* < 0.0001, Figure 2A). The median (IQR) pH values were 5.95 (5.50–7.47), 6.51 (5.62–7.22), and 7.08 (5.41–7.83) at the ear pinnae, axillae, and inguinal region, respectively, and the highest values were observed at the inguinal region (*p* = 0.035, Figure 2B). The median (IQR) sebum levels (μg/cm^2^) were 14.00 (1.60–25.50), 2.92 (2.24–5.80), and 2.64 (1.60–5.26) at the ear pinnae, axillae, and inguinal region, respectively, and the highest values were observed at the ear pinnae (*p* = 0.028, Figure 2C). The median (IQR) temperature values were 36.62 (36.02–37.89), 36.44 (36.32–36.79), and 36.48 (36.08–37.70) at the ear pinnae, axillae, and inguinal region, respectively, and no significant differences were observed (*p* = 0.486, Figure 2D). The median (IQR) TEWL values (g/m^2^/h) were 10.18 (7.03–14.00), 12.26 (8.00–17.40), and 8.48 (5.82–17.66) at the ear pinnae, axillae, and inguinal region, respectively, and no significant differences were observed (*p* = 0.312, Figure 2E). Significant site-related differences were observed in skin hydration, pH, and sebum. However, temperature and TEWL showed no site-dependent variations.

### 3.3 Interaction between age and site

**Figure 3:**
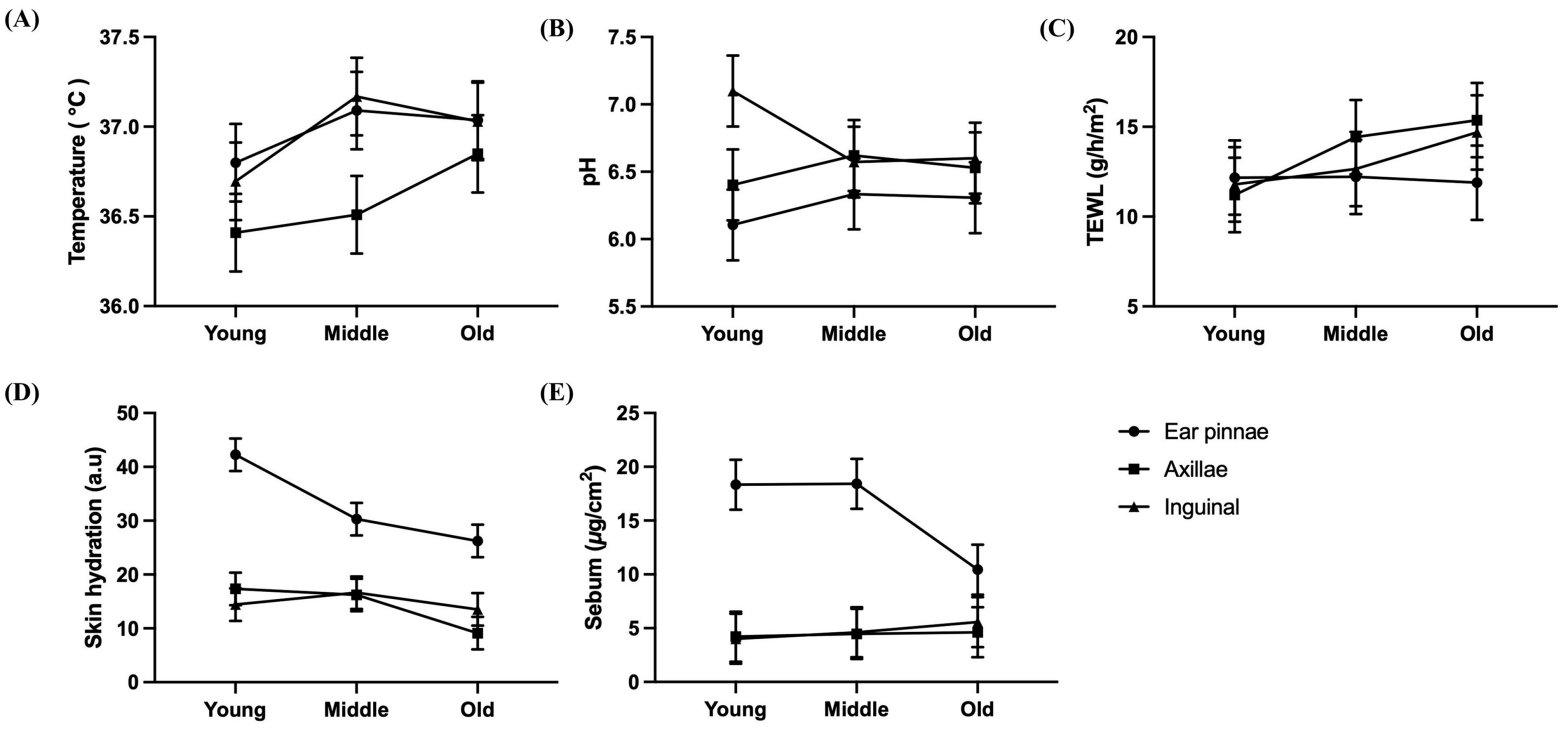
Age and site variations in skin barrier parameters for visualizing interaction patterns. **(A)** Skin temperature, **(B)** pH, **(C)** transepidermal water loss, **(D)** skin hydration, and **(E)** sebum. Data are presented as the mean ± standard error of the mean to illustrate overall trends. Statistical analyses based on median values and nonparametric tests are presented in Figures 1, 2.

Figure 3 shows the interaction patterns between age and anatomical site. Although variations among age groups appeared to differ by site, no separate statistical test for interaction was performed. Thus, Figure 3 is provided for descriptive visualization only.

### 3.4 Correlations between skin barrier parameters

**Figure 4:**
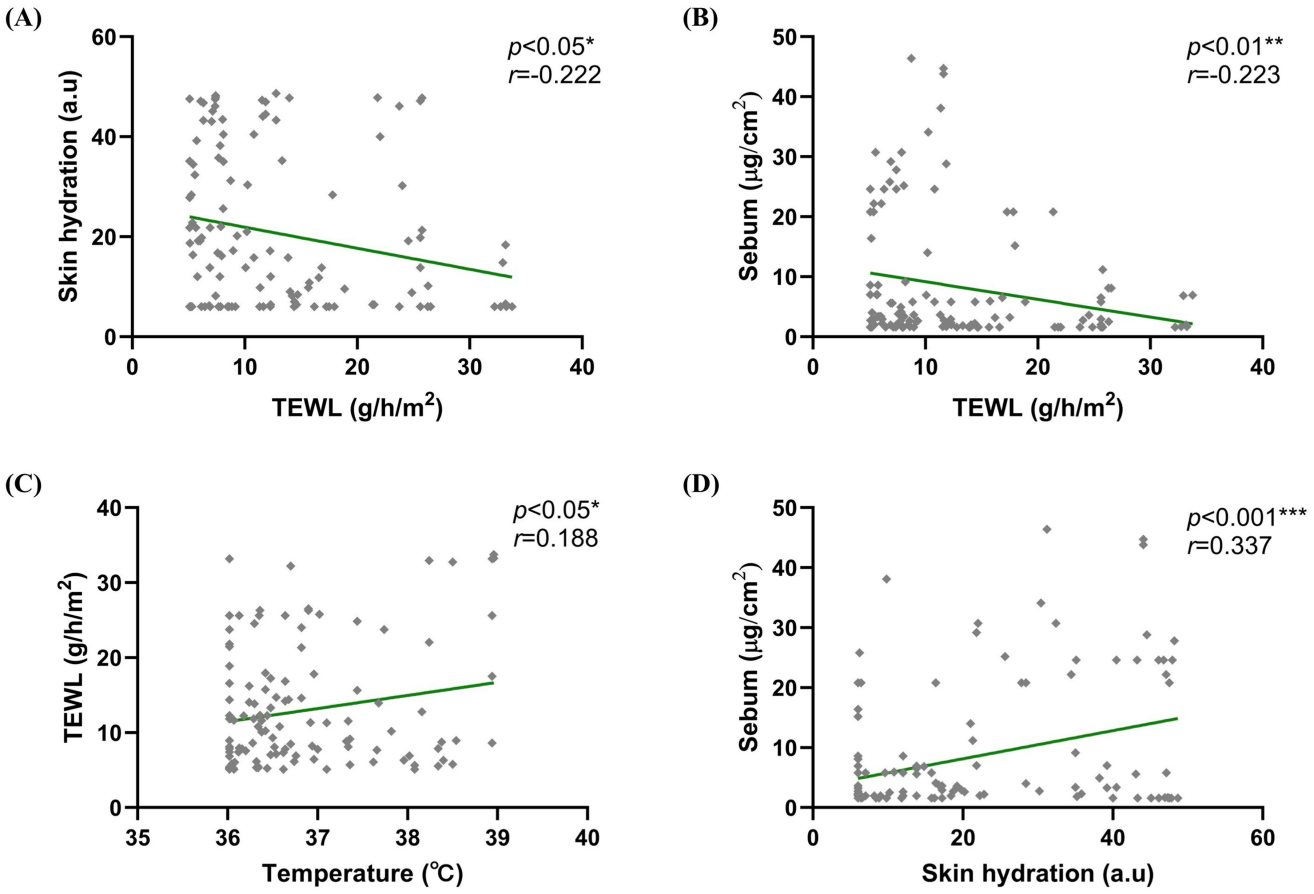
Correlation between skin barrier parameters. Negative correlations were observed between **(A)** TEWL and skin hydration (*p* = 0.010; *r* = −0.222) and between **(B)** TEWL and sebum levels (*p* = 0.009; *r* = −0.223). Meanwhile, positive correlations were observed between **(C)** TEWL and temperature (*p* = 0.029; *r* = 0.188) and between **(D)** sebum and skin hydration (*p* < 0.001; *r* = 0.337). Pearson’s correlation coefficients are presented.

Pearson’s correlation analysis was used to evaluate correlations between skin barrier parameters. TEWL was negatively correlated with skin hydration (*p* = 0.010; *r* = −0.222) and sebum (*p* = 0.009; *r* = −0.223) and positively correlated with temperature (*p* = 0.029; *r* = 0.188). A significant positive correlation was also observed between sebum and skin hydration (*p* < 0.001; *r* = 0.337). No other significant correlations were found. Figure 4 shows the scatter plots of significant correlations.

In age-specific analyses, the young group showed positive correlations between temperature and TEWL (*p* = 0.023; *r* = 0.338) and between skin hydration and sebum (*p* = 0.001; *r* = 0.491) and a negative correlation between skin hydration and pH (*p* = 0.402; *r* = −0.305). Meanwhile, in the middle-aged (*p* = 0.023; *r* = −0.338) and old groups (*p* = 0.007; *r* = −0.398), a negative correlation was observed between skin hydration and TEWL (data not shown). No other significant correlations were found within the age groups.

### 3.5 Breed comparison

In this study, 45 retrievers (nine golden retrievers and 36 labrador retrievers) were included, with 15 dogs in the young, middle-aged, and old groups (Supplementary Table 1). There were no significant differences between breeds in age- or site-specific skin barrier parameters. However, in the old group, the inguinal skin temperature was significantly higher in labrador retrievers than in golden retrievers (*p* = 0.003, Supplementary Tables 2, 3).

## 4 Discussion

In this study, the skin barrier parameters were evaluated across age groups and anatomical sites in healthy retrievers, and correlations between these parameters were further analyzed. TEWL, which reflects the amount of water evaporating through the stratum corneum, is widely recognized as a representative indicator of skin barrier function (21). Skin hydration represents the stratum corneum’s water content, which is maintained by natural moisturizing factors and intercellular lipids (22). The skin surface pH plays an important role in preventing microbial invasion, stabilizing the lipid matrix, and regulating enzyme activity, whereas sebum contributes to reducing water loss and forming a chemical barrier against pathogens (5, 23). The skin temperature reflects local blood flow and metabolic activity and can also be correlated with TEWL (24). In the present study, these noninvasive parameters were used to characterize variation across age and anatomical sites and to evaluate relationships among them.

Skin hydration was significantly reduced in older dogs, which is consistent with a previous human study reporting age-related decreases in stratum corneum hydration (25). In humans, a decline in ceramides and other lipids in the stratum corneum is considered a major mechanism for reduced water retention (26). A similar age-related decrease in ceramides has been reported in dogs, supporting the findings of the present study (27). Although skin hydration decreased with age in dogs, TEWL tended to increase, consistent with previous reports (15). Age-related changes in TEWL have been described in humans, but they vary according to the site, sex, and skin type (25). Similarly, canine studies have reported TEWL variations depending on the site, breed, and age (17, 28). The closed-chamber TEWL device used in the present study may have lower sensitivity than open-chamber systems for detecting subtle site-related differences (29). However, closed-chamber devices are less affected by environmental fluctuations such as temperature, humidity, and air flow, which is a recognized advantage of this method (30). To enhance sensitivity for detecting fine anatomical differences, future studies would benefit from comparative evaluations using different chamber systems. Although a previous study has observed increases in skin pH and decreases in sebum with aging in humans (6), these changes were not observed in the present study. This discrepancy may reflect the limited age distribution in this study, particularly the absence of geriatric dogs (31), or breed-specific characteristics of the study population. In addition, the cross-sectional design did not allow for the evaluation of longitudinal changes over time, limiting interpretation of true age-related progression.

Site-related differences in hydration, pH, and sebum levels were identified. The ear pinnae showed higher hydration and sebum levels than other regions, whereas the inguinal region exhibited the highest pH. In humans, increased inguinal pH has also been reported in apocrine-gland-rich sites, such as the axillae and groin. However, as apocrine glands are distributed throughout canine skin, this mechanism may not be directly applicable (15, 23). The higher pH in the inguinal region may reflect a local occlusion effect, as increased humidity, temperature, and reduced oxygen tension in occluded areas are known to disrupt the acid mantle in humans (32), and similar conditions may occur in dogs. Higher pH values have also been reported in dogs in folded or densely haired regions such as the abdomen and groin (16, 33). The elevated sebum levels at the ear pinnae resemble findings in humans at sebaceous-gland-rich areas, including the scalp and face (34), and may be associated with the anatomical distribution of sebaceous glands in the auricular region (35). The elevated hydration at the ear pinnae are consistent with previous findings (17), although the underlying mechanism remains unclear. By contrast, no significant site-related differences were observed in TEWL or skin temperature. Given that the skin microbiome strongly influences pH, hydration, and lipid metabolism (36), the absence of microbiome profiling in the present study limits the interpretation of these site-specific differences, highlighting the need for further research in dogs.

Most skin barrier parameters did not significantly differ between golden retrievers and labrador retrievers, suggesting that skin barrier characteristics are largely consistent within the retriever group. A previous study comparing different breeds reported differences in skin parameters (15). However, to the best of our knowledge, this study is the first to compare parameters between sub-breeds within the retriever group. A difference in inguinal skin temperature was observed in the old group, yet the limited and unbalanced breed distribution warrants cautious interpretation, and confirmation in larger, breed-balanced study will be necessary. The devices used in this study have been widely used in canine studies for noninvasive skin assessment (15, 16, 22, 29). However, considerable variability has been reported for certain measurements, including sebum levels (3). Because hair density and length may influence biophysical parameters (3, 16), we minimized hair-related interference by selecting the least-haired areas at each anatomical site. Future studies should adopt standardized methodologies that account for coat characteristics and more rigorously evaluate reproducibility across multiple skin sites.

Furthermore, the positive correlation observed between hydration and sebum suggests that sebum secretion may contribute to maintaining stratum corneum hydration. TEWL was negatively correlated with hydration and sebum, indicating that reduced stratum corneum hydration and diminished lipid content may contribute to increased water loss, consistent with previous human findings (37). Although no study has demonstrated a significant correlation between TEWL and sebum in dogs (17), reduced sebum production may exacerbate skin barrier dysfunction by diminishing the skin’s ability to limit evaporation (4, 5). A previous study noted variability in the relationship between TEWL and hydration depending on the site and measurement conditions (38). Nevertheless, experimental evidence suggests that increased hydration can reduce TEWL (37), highlighting the interaction between stratum corneum hydration and barrier function. The positive correlation between hydration and sebum reflects the interplay between the water content in the stratum corneum and maintenance of the lipid layer, a relationship that has also been documented in human studies (6). Together, these findings suggest that sebum contributes to maintaining stratum corneum hydration. TEWL also showed a positive correlation with skin temperature, a finding that is consistent with a previous study in humans (7). Although animals were acclimated for 15 min under standardized indoor conditions with controlled temperature and humidity, seasonal variation and outdoor environmental temperature were not fully controlled in this study. Because skin temperature is highly sensitive to ambient factors such as temperature and humidity (10, 39), the interpretation of these findings requires caution.

The results of age-stratified analyses showed some correlations within groups, but interpretation is limited by the small sample sizes. In addition, site-specific relationships could not be fully clarified because data from the three sites were pooled for correlation analysis, indicating the need for future studies with larger sample sizes and site-specific evaluations. Furthermore, Future studies that include repeated-day or intra-observer assessments would help further validate the consistency of these biophysical measurements.

This study provides baseline data on skin barrier parameters in a healthy, single-breed canine population. The indicators evaluated in this study, including hydration, TEWL, pH and sebum, are considered fundamental components of epithelial barrier homeostasis. The epithelial barrier theory proposes that these elements can exhibit physiological variability and be modulated by environmental exposures, immune responses and microbial composition (40). From this perspective, the age and site-related differences identified here demonstrate that these basic barrier markers can vary according to individual characteristics and anatomical regions, and this provides important comparative baseline data for future investigations assessing the effects of external factors on canine skin barrier function. Furthermore, the present study extends previous research by evaluating multiple anatomical sites and age groups simultaneously. The correlation analyses provide significant insights into the interactions among these parameters, thereby supporting the interpretation of complex alterations to barriers observed in diseased states.

## 5 Conclusion

This study provided baseline data for age- and site-related variations in skin barrier parameters and their correlations in healthy retrievers. These findings may serve as reference values in canine dermatological research and provide a basis for interpreting alterations in disease conditions, particularly because they were derived from multiple anatomical sites and age groups in healthy dogs.

## Data Availability

The original contributions presented in the study are included in the article/Supplementary material, further inquiries can be directed to the corresponding author.
